# Real-time PCR in Food Science: Current Technology and Applications

**DOI:** 10.3201/eid.1908.130524

**Published:** 2013-08

**Authors:** Deborah F. Talkington

**Affiliations:** Centers for Disease Control and Prevention, Atlanta, Georgia, USA

**Keywords:** PCR, real-time PCR, quantitative PCR, qPCR, food safety, food quality, food microbiology, enteric infections, food science

This 302-page book describes methodologies and applications of real-time PCR in food science. In addition to detection of enteric pathogens, including foodborne and waterborne parasites, a section on food quality provides information on the use of this method to detect genetically modified organisms, allergens, and animal or plant species in food products. This book will be valuable to food scientists with an interest in real-time PCR, also known as quantitative PCR or qPCR (not to be confused with reverse transcription PCR, or RT-PCR). This book provides a comprehensive overview of conventional and qPCR methods used to detect pathogens in contaminated foods, as well as their use in analysis of food integrity, including the detection of genetically modified organisms, allergens, and authentication of biological species in labeled foods. The book is divided into 3 sections.

The first section, comprising 4 chapters, provides a detailed examination of basic methodologies of qPCR. The inclusion of an introduction to these methodologies, sample preparations, assay design, and the role of controls is beneficial for new scientists and experienced readers. As a bonus, color plates are included.

The second and third sections span 14 chapters. Each of the chapters covering detection of enteric pathogens is organized similarly, which allows the reader to quickly compare and locate information. Bacterial pathogens are discussed in separate chapters; viruses and foodborne and waterborne parasitic pathogens are covered in 2 comprehensive chapters. Each chapter includes background information about organism types, tables of published assays, current methods, the use of controls, approaches to determine limits of detection, and current challenges. A compelling chapter on standardization of qPCR methods compares the International Organization for Standardization and the European Organization for Standardization platforms, giving the reader a glimpse into international processes for creating standards.

Rounding out this text are chapters focused on the use of qPCR to detect allergens, gluten, and genetically modified organisms and chapters addressing authentication of animal or plant species present in labeled foods. This book provides an excellent, detailed guide for anyone interested in development and use of qPCR platforms for food safety, quality, and microbiology.

